# Intermittent Theta Burst Stimulation (iTBS) as an Optimal Treatment for Schizophrenia Risk Decision: an ERSP Study

**DOI:** 10.3389/fpsyt.2021.594102

**Published:** 2021-05-10

**Authors:** Yang Wu, Lu Wang, Fengqiong Yu, Gong-Jun Ji, Guixian Xiao, Xu Feifei, Zhu Chunyan, Chen Xingui, Kai Wang

**Affiliations:** ^1^School of Mental Health and Psychological Sciences, Anhui Medical University, Hefei, China; ^2^Department of Neurology, First Affiliated Hospital, Anhui Medical University, Hefei, China; ^3^Institute of Artificial Intelligence, Hefei Comprehensive National Science Center, Hefei, China; ^4^Anhui Province Key Laboratory of Cognition and Neuropsychiatric Disorders, Anhui Medical University, Hefei, China; ^5^Collaborative Innovation Center for Neuropsychiatric Disorders and Mental Health, Hefei, China

**Keywords:** schizophrenia, intermittent theta-burst stimulation, risk decision-making, Iowa Gambling Task, ERSP

## Abstract

**Objective:** People with schizophrenia have serious impairments in social function, especially in decision-making ability. Transcranial magnetic stimulation modified intermittent theta burst transcranial magnetic stimulation (iTBS) has been shown to regulate the functional connection of brain networks. Our study explored the therapeutic effect of iTBS on decision-making disorders in schizophrenia.

**Methods:** Participants were pseudorandomized and assigned to iTBS (*n* = 16) or sham (*n* = 16) group. iTBS group was administered 1,800 pulses on the target of the left dorsol lateral prefrontal cortex (L-DLPFC) per day for 14 consecutive days. We compared Iowa gambling task performance and associated event-related spectral perturbation results (ERSP) among two groups.

**Results:** The results show that participants' performance in the high-lose in the iTBS group had stronger stimulation of theta spectral power than those in the sham group. Specifically, we found that under high-risk conditions, compared with the control group, the iTBS group showed significant activation of the theta spectrum power in the FPZ, FZ, FCZ, and CZ regions after treatment.

**Conclusions:** Our results provide evidence that long-term iTBS stimulation effectively improves the decision-making ability of schizophrenia. After receiving negative feedback, patients can turn to safety options. These findings support that iTBS may be a potential treatment for clinical decision-making disorders.

## Highlights

- The modulation of DLPFC by intermittent theta burst transcranial magnetic stimulation (iTBS) changed the risk decision.
- iTBS can induce the left dorsolateral prefrontal lobe to show stronger theta-band activity.
- iTBS has a special advantage in improving the risk decision-making of patients with schizophrenia.

## Introduction

Patients with schizophrenia exhibit not only positive and negative symptoms but also, generally, varying degrees of social cognitive impairment (1). Decision-making function is an important component of social cognitive function; it is also an important indicator of executive function (2). According to previous research, the decision-making ability of patients with schizophrenia is severely impaired (3, 4). During a task, they cannot adjust the subsequent choices according to the results of previous choices and either cannot choose or need to spend a long time choosing the favorable options. Compared with low-risk and long-term favorable options, patients with schizophrenia are more inclined to choose high-risk and long-term unfavorable options (5–7). Currently, clinical antipsychotic drugs are not effective in improving social cognitive deficits, and new treatment methods are urgently needed to solve the decision-making obstacles of patients with schizophrenia (8).

Brain imaging studies indicate that the trade-off between risks and benefits in the decision-making process involves a complex neural network that includes brain regions such as the dorsal lateral prefrontal cortex (DLPFC), medial prefrontal cortex (mPFC), anterior island lobe, and anterior cingulate cortex; however, the causal effect of this network on risk decision-making remains unclear (9–11). Increasing evidence indicates that the frontal cortex is involved in the continuous internal monitoring of movements and is critical in situations related to response control (12). Resting neuron activity in the DLPFC is related to preference formation induced by individual selection. The left DLPFC (L-DLPFC) in the human brain plays an important role in the process of transforming basic social signals into self-directed decision-making (13). These studies further demonstrate the close association between frontal regions and decision-making processes. In addition, it was also found that the decision-making ability of patients with schizophrenia is impaired, and they show obvious defects in the application of decision-making rules, which may lead to poor suppression control. Therefore, the frontal cortex is a critical area in the decision-making process, and regulation of prefrontal function may help improve the decision-making function of patients with schizophrenia.

Transcranial magnetic stimulation (TMS) is a non-invasive biological stimulation technology that induces long-term changes in the excitatory and inhibitory activities of the target network according to different modes of stimulation (14). To date, TMS has been used to study decision-making behavior. Previous research has confirmed that TMS induces the human brain and can help individuals avoid risky decision-making behaviors (15–17). As a new mode of TMS, intermittent theta burst stimulation (iTBS) may have a better promoting effect than that by the traditional stimulation mode on the regulation of cortical excitability of the brain regions related to decision-making (18). iTBS consists of clusters of burst stimulation to excite the cerebral cortex, which affects brain metabolism and the electrical activity of nerves, with the advantages of no pain or damage, and shorter treatment time (19, 20). In addition, previous studies have proved the effectiveness of iTBS in interfering with the symptoms of schizophrenia (21). Thus, iTBS combined with magnetic resonance imaging (MRI)-guided navigation would have greater efficacy and may be a preferred strategy for the treatment of decision-making disorders in schizophrenia.

The decision-making process occurs within a few 100 milliseconds; hence, methods with high temporal resolution, such as event-related spectral perturbation (ERSP), can be used to better study this process (22). Owing to the strong oscillation of the electrophysiological activity of the brain, it is easy to lose a large amount of cognitive-related electroencephalography (EEG) information in the time-locked average (23). To overcome this problem, ERSP technology is more helpful in exploring the evaluation process in ambiguous situations. This is a suitable method for evaluating the time-frequency characteristics of EEG. It is used to evaluate the change in the average EEG power spectrum in a certain frequency band. The greatest advantage of ERSP is that it can be used to reflect cognitive processes that traditional event-related potential (ERP) technology cannot reflect (24, 25). This technique has been proven effective in exploring the cognitive dynamics of decision-making, and its results can be used as an indicator of the effectiveness of behavior prediction (26). In the time-frequency domain, theta power (4–7 Hz) is an indicator of the brain's decision-making process (27). Therefore, the dynamics of theta activity will reflect the process of risk, average return, and integration between the two.

The Iowa Gambling Task (IGT) is a classic experimental paradigm used to examine decision-making behavior and has been proven useful in detecting decision-making disorders in various neurological and psychiatric disorders (28–30). Therefore, the purpose of this study was to investigate whether the application of iTBS to the L-DLPFC of patients with schizophrenia would change their risk attitudes in ambiguous situations, and to reflect this result by comparing the brain electrical responses in IGT tasks before and after stimulation. The following hypotheses were made: (1) in high-risk decisions, prefrontal theta-band neural oscillations induced by real stimuli are stronger than those induced by sham stimuli; (2) compared with sham stimulation, iTBS could effectively improve the decision-making ability of patients with schizophrenia.

## Materials and Methods

### Subjects

The study initially recruited 36 right-handed participants aged 17–52 years, all from the clinic of the Anhui Mental Health Center in Hefei, China. Four subjects were disqualified due to personal reasons or refusal to complete the scan. In the end, 32 participants successfully completed the test. These participants were randomly assigned into two groups: the real and sham stimulation groups (*n* = 16 in each). All patients were diagnosed by two psychiatrists based on structured clinical interviews in the fourth edition of the Manual of Diagnosis and Statistics of Mental Disorders (SCID-IV). Before the study, all subjects received a stable dose of atypical antipsychotic medication including risperidone, chlorpromazine, and olanzapine, while the remaining types of drugs would be converted into equal doses of olanzapine. The total Positive and Negative Syndrome Scale (PANSS) was used to assess the severity of symptoms in patients with schizophrenia. Exclusion criteria were obvious head trauma with loss of consciousness, history of neurological disease or drug abuse, Hamilton Anxiety Rating Scale (HAMA) or Hamilton Depression Rating Scale (HAMD) score >14 points, and head motion exceeding 3 mm in translation or 3° in rotation during resting-state functional MRI scanning. The study was registered at www.clinicaltrials.gov (NCT03868358) and was approved by the Ethics Committee of Anhui Medical University. All patients or their guardians signed an informed consent form before the experiment.

### Neuro-navigated Transcranial Magnetic Stimulation

To obtain the stimulation target, the L-DLPFC, all participants underwent MRI brain scans before the study. The target was regarded as a spherical image centered at the superficial central point of the L-DLPFC in the standard brain template [Montreal Neurological Institute (MNI): −38, 44, 26; radius: 6 mm] (31), and based on the T1-weighted anatomic magnetic resonance structure image, the target of the L-DLPFC was transformed into the brain structure image of each individual subject by the SPM (www.fil.ion.ucl.ac.uk/spm) and TMStarget softwares (32). This target was then introduced into the frameless neuronavigation system (Visor 2.0, Advanced Neuro Technologies). The coil was maintained horizontally and tangentially to the skull pointing forward, and its central point overlapped with the target of the L-DLPFC (32, 33). A frame-free stereoscopic optical tracking and navigation system was applied for positioning. The entire treatment process was monitored dynamically and in real time to ensure accuracy of the target.

iTBS was administered at 1,800 pulses per day for 14 consecutive days (Supplementary Figure 1), from the 2nd to the 14th day. iTBS treatment used a MagStim Rapid2 stimulator (MagStim Company Ltd.) with a 70-mm air-cooled figure-of-eight coil. Each session of iTBS lasted 190 s and consisted of three pulses transmitted at 50 Hz, which was repeated every 200 ms (at 5 Hz) for a total of 600 pulses (34). According to previous methodological research, this 190-s protocol was repeated thrice (1,800 pulses in total) to obtain cumulative aftereffects, with 15 min between each session (controlled by a stopwatch) (35–37). According to the five-step procedure (38), the resting motor threshold (RMT) was measured at each visit and iTBS was delivered at 80% of the RMT (39). During the treatment, all patients sat comfortably in chairs and wore silencer earplugs to prevent hearing damage. During the 15-min treatment interval, all patients remained silent and closed their eyes to rest (35).

The placebo treatment had the same treatment regimen and duration as those of the real iTBS group; however, while the coils used looked the same and produced the same sounds, no magnetic impulses were given.

### Task and Procedure

Before iTBS treatment, the PANSS, Scale for the Assessment of Positive Symptoms (SAPS), and Scale for the Assessment of Negative Symptoms (SANS) were administered by a trained investigator to assess the baseline severity of the patients' symptoms. In addition, participants performed a modified version of the Iowa game task (IGT) (40) to assess their decision-making propensity in ambiguous situations. On the last visit, the PANSS, SAPS, and SANS were administered again to assess the overall treatment efficacy. All experimental stimuli were presented on a 17-inch flat-screen CRT color display. At the beginning of each trial, a two-digit selection stimulus, with two digital betting points (50-left box, 100-right box), represented the monetary values in RMB. Participants were informed that the initial amount was 1,000 Yuan; based on this amount, they should aim to win as much as possible. The winning and losing rates were set in advance. Of the bets, bets of 50 Yuan were set to a 60% win rate and bets of 100 Yuan were set to a 40% win rate; that is, 50 is a favorable option and 100 is an unfavorable option. However, winning or losing was random, and the participants were not informed of the odds. After choosing to place a bet, a blank screen appeared for 200–400 ms (with a “+” gaze point in the center of the screen), followed by a cartoon face (a smiling face represents winning money, positive feedback; a depressed face represents losing money, negative feedback). This lasted for 1,000 ms, and the text and numerical letter appeared for 1,000 ms at the end to inform the participants of the result of this bet. At the end of a trial, the selection stimulus reappeared, and the next trial began. The task consisted of three parts, each with 100 trials, and a total of 300 trials. In the behavioral statistics, 300 trials were divided into six blocks on average. Behavioral indicators were as follows: the number of times to choose 50 (favorable option) minus the number of times to choose 100 (bad option) for each interval; the higher the net score, the better the performance (Supplementary Figure 2).

### Electrophysiological Recordings

A modified version of the IGT was used to assess decision-making propensity in ambiguous situations while recording changes with EEG (40). The NeuroScan ERP recording system (Neuro Scan, Sterling, VA, USA) was used to record the EEG data of 64 scalp elastic caps, which were expanded according to the international 10–20 system. During the EEG recording, each electrode was referenced to the left mastoid. When recording online, the scalp resistance of each electrode was kept below 10 kΩ. The filtered wideband for recording was 0.05–100 Hz. EEG was continuously sampled at a sampling frequency of 500 Hz. After the EEG data were recorded, it was processed offline through MATLAB software scripts and the EEGLAB toolbox. Digital filtering for offline analysis was performed with a low-pass 30-Hz filter. Trials with a signal exceeding ± 100 mV were excluded from averaging to eliminate electrooculograms (EOG) and movement artifacts. Independent component analysis (ICA) was performed using the EEGLAB toolbox and components including blinks, eye movements, electromyography, and other artifacts were removed from the EEG data. The artificial components of electrooculogram and electromyogram were identified and removed by the EEG_SASICA plug-in in EEGLAB. On average, each participant has 41.09 [95%, (40, 42)] components remaining. The mean proportion of rejected epochs was 9.75% [95%, (6, 13)] in pre_iTBS group, 13.98% [95%, (7, 21)] in pre_sham group, 13.98% [95%, (7, 21)] in post_iTBS group and 15.81% [95%, (8, 23)] in post_iTBS group. Rejection rates did not differ significantly among groups (*F* = 0.909, *p* = 0.441).

#### Statistical Analysis

All statistical analyses were performed using SPSS (version 17; Chicago, IL, USA). For statistical analysis of behavioral data, this study defines the total net score as the difference between the numbers of low- and high-yield options. The entire test procedure is divided into six blocks in equal order, and each block contains 50 tests on average. Two-factor repeated-measures analysis of variance (ANOVA) was used to evaluate the difference in value between good and bad choices, using blocks as internal factors for the subjects and groups as factors between the subjects. ERSP results were analyzed using multivariate repeated-measures ANOVAs with feedback type (loss and win), intensity (50 as low condition and 100 as high condition), electrode (FPZ, FZ, FCZ, and CZ) and time as within-subject factors and group (iTBS group and sham group) as the between-subject factor (41). To better understand the efficacy of iTBS treatment on patients with schizophrenia, we also used a paired-sample *t*-test to analyze the performance of the iTBS and sham groups in the IGT task after treatment. In addition, we calculated the correlation between clinical behavior and symptom scores (Detailed results are in the supplement).

## Results

### Demographic, Clinical, and Neuropsychological Assessments

Demographic and neuropsychological data are summarized in Table 1. There was no significant difference between the two groups in sex (*t* = 1.296, *p* = 0.202), age (*t* = −1.296, *p* = 0.214), or education level (*t* = −0.648, *p* = 0.522). Similarly, there was no significant difference in HAMA, HAMD, PANSS, SAPS, or SANS scores at baseline between the iTBS and sham groups (all *p* > 0.05). We found that PANSS (including PANSS Total, PANSS Positive, PANSS Negative, PANSS General), SAPS, and SANS scores showed significant improvement over time. In addition, a mixed-design (2 × 2) ANOVA showed the “group × time” interactions to be significant. As shown in Table 1, the total scores of PANSS (*F* = 7.455, *p* = 0.010) and SANS (*F* = 14.592, *p* = 0.001) were significantly reduced.

**Table 1:** Planned 2 × 2 repeated measure ANOVA on over time (pre-TMS, post-TMS).

	**iTBS treatment (*****n*** **=** **16)**		**Sham treatment (*****n*** **=** **16)**		**Baseline comparison**		**Factor time**		**Group by time interaction**		
	**Pre**	**Post**	**Pre**	**Post**	***t***	***p***^**a**^	***F***	***p***^**b**^	**F**	***p***^**b**^	**Effect sizes**^**c**^
**Demographic indictors**											
Gender (M/F)	26.56(1.70)	NA	25.72(2.76)	26.52(2.69)	1.296	0.202	NA	NA	NA	NA	NA
Age (years)	22.06(3.33)	NA	26.06(9.69)	NA	−1.296	0.214	NA	NA	NA	NA	NA
Education (years)	12.02(2.49)	NA	12.63(2.41)	NA	−0.648	0.522	NA	NA	NA	NA	NA
**Emotion**											
HAMA	4.56(2.03)	2.94(1.48)	5.88(3.32)	5.06(5.50)	−1.348	0.188	2.994	0.094	0.333	0.568	0.011
HAMD	4.06(2.17)	3.31(1.35)	4.38(3.79)	5.50(3.67)	−1.201	0.239	0.396	0.534	0.776	0.385	0.025
**Clinical characteristics**											
PANSS total	60.44(14.13)	49.81(10.82)	58.75(9.98)	54.56(11.84)	0.390	0.699	36.469	0.000^***^	7.455	0.010^*^	0.100
PANSS positive	13.06(5.07)	10.25(3.00)	13.06(4.14)	11.25(3.89)	0.000	1.000	24.816	0.000^***^	1.160	0.290	0.037
PANSS negative	16.38(3.61)	13.50(4.40)	15.00(4.63)	13.63(4.65)	0.936	0.357	20.304	0.000^***^	2.529	0.122	0.078
PANSS general	27.06(6.91)	22.56(5.15)	26.88(5.06)	25.13(6.50)	0.088	0.931	22.700	0.000^***^	4.395	0.045	0.128
SAPS	25.50(10.23)	20.63(7.42)	32.13(14.29)	29.63(11.47)	−1.508	0.142	20.598	0.000^***^	2.136	0.154	0.006
SANS	39.44(12.77)	29.31(8.71)	39.56(12.69)	37.56(10.81)	−0.028	0.978	32.497	0.000^***^	14.592	0.001^**^	0.329
Illness duration (years)	3.43(3.16)	NA	3.67(4.07)	NA	−0.194	0.847	NA	NA	NA	NA	NA
Age at onset (years)	7.64(0.89)	7.47(1.03)	7.72(0.98)	7.76(0.78)	−0.316	0.754	0.344	0.560	0.938	0.338	0.019
Olanzapine equivalent (mg)	4.90(1.45)	5.00(1.22)	4.83(1.37)	4.87(1.42)	0.169	0.866	0.025	0.874	0.180	0.673	0.004

*M/F, male/female*.

a *Two-sample t-test between pre- real and pre- sham TMS treatment*.

b *Group by time interaction effect by repeated measures ANOVA*.

c *Effect sizes for the interaction between group and time of measurement were calculated by subtracting the mean score post treatment from the mean score before treatment for each group, subsequently determining the difference between the 2 groups (iTBS, sham) and then dividing the results by the pooled SDs*.

* *p < 0.05*.

** *p < 0.01*.

*** *p < 0.001*.

### Behavioral Results

**Figure 1:**
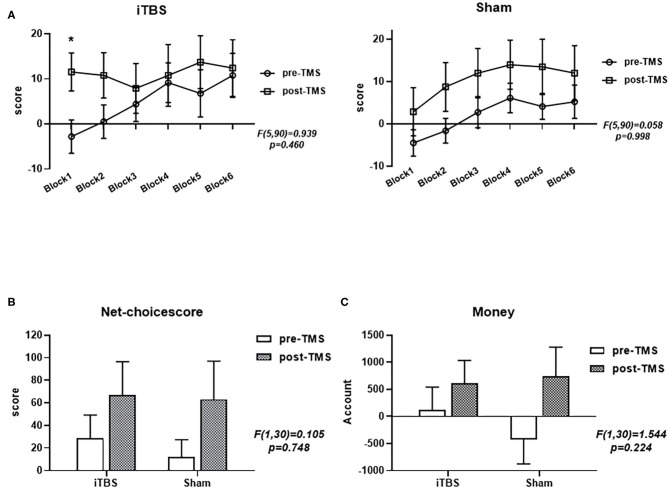
Performance of the intermittent theta burst stimulation (iTBS) and sham groups in the revised Iowa Gambling Test (IGT). Comparisons of the two groups' net scores on the six blocks **(A)**, final total net choice scores **(B)**, and final monetary amount **(C)** before and after treatment. ^***^p < 0.001; ^**^p < 0.01; ^*^p < 0.05; ns: *p* > 0.05.

A multivariate analysis was performed to examine the relationship between IGT performance and time in each group (Figure 1). We analyzed the performance of IGT using mixed-design (2 × 2) ANOVA, which used a “group × time” interaction to examine the relationship between group and time, including net scores and remaining money (Figures 1B,C). In terms of net scores (*F* = 0.105, *p* = 0.748) and remaining amount (*F* = 1.544, *p* = 0.224), there was no significant difference in the interaction between the group and the measured time-point (pre-TMS, post-TMS). We further analyzed the main effect of time, and the net scores of both groups showed an increasing trend as the task progressed (Figure 1A). Both the net score (*F* = 5.146, *p* = 0.031) and the remaining money (*F* = 9.027, *p* = 0.005) showed significant time main effects. In addition, we also compared the net scores of the two groups from six blocks of the two groups before and after treatment. Neither group (iTBS, *F* = 0.929, *p* = 0.460; sham, *F* = 0.058, *p* = 0.998) showed a significant difference. However, the net score of the iTBS group on Block1 increased significantly after treatment.

### Event-Related Spectral Perturbation Results

**Figure 2:**
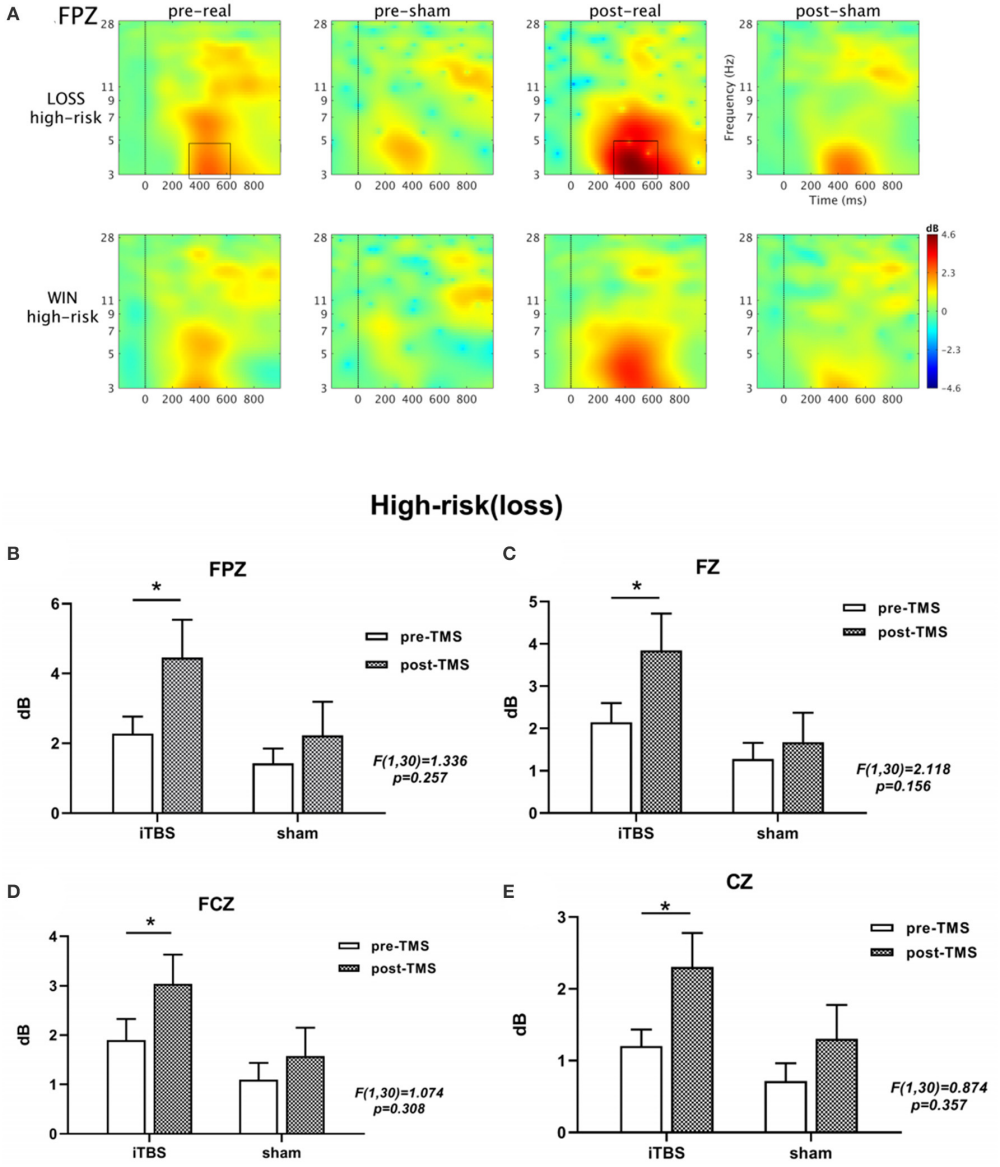
The results obtained from the FPZ electrode is shown in **(A)**. A significant difference in ERSP is seen between the two conditions (winning and losing) before and after treatment under high-risk conditions based on a paired t-test. The black boxes define the time-frequency region of interest where the power increases significantly. In addition, **(B-E)** show the *θ*-band active bar graph at four locations. ^***^p < 0.001; ^**^p < 0.01; ^*^p < 0.05; ns: *p* > 0.05.

In the time-frequency analysis, we selected 4 electrode points (including FPZ, FZ, FCZ and CZ) when the stimulus lock was activated, and calculated the neural oscillations at the 4 electrode points, respectively. Based on the results of previous literature and the results of the overall average of the stimulus pictures, we focused on analyzing the time window of 400–550 ms after the picture is presented, and the corresponding ERSP frequency was 3-5 Hz. The multivariate repeated-measures ANOVAs revealed significant main effects of feedback type (*F* = 17.446, *p* = 0.000), as well as marginal significant main effects of electrode (*F* = 2.917, *p* = 0.052) and time (*F* = 3.495, *p* = 0.071). Under high-risk and loss conditions, the activation of FPZ site is more obvious after treatment (Figure 2 and Supplementary Figure 3). As shown in Supplementary Table 1, we also used a mixed-design two-way ANOVA to analyze the four electrode points under the two conditions of winning and losing, indicating theta frequency neural oscillation activity. No significant group × time (2 × 2) interaction effect was found (all *p* > 0.05). In addition, we also used paired-sample *t*-tests to analyze the performance of the iTBS and sham groups in the IGT task after treatment. Further analysis results showed that at the FPZ, FZ, FCZ, and CZ electrode points, there was a significant difference in the theta frequency nerve oscillation activity before and after stimulation in the iTBS group (FPZ: *p* = 0.030; FZ: *p* = 0.023; FCZ: *p* = 0.034; CZ: *p* = 0.016) (Figure 2 and Supplementary Figure 3). No significant differences were found at any electrode point in the sham group (all *p* > 0.05). The results show that in these four sites participants' performance in the high-loss in the iTBS group had stronger stimulation of theta spectral power than those in the sham group, and the activation at the FPZ site was the most obvious.

## Discussion

In our study, we compared the effects of a 2-week intervention on the decision-making ability of patients with schizophrenia, including behavioral and electrophysiological measurements. In terms of improving the decision-making ability, the iTBS group had particular advantages and relatively stable clinical effects. The behavioral results showed that over time, the decision-making ability of both groups improved; however, there was no significant difference between before and after treatment. However, in the ERSP results, the loss of 100 Yuan in the iTBS group showed an enhancement of theta-band activation in the 400–550 ms region relative to the sham group. In other words, our results showed that iTBS offers significant advantages in decision-making, and is effective in improving the decision-making ability of patients with schizophrenia. Overall, this study provides new evidence for the feasibility of TMS in improving decision-making abilities in patients with schizophrenia.

The IGT is an ambiguity decision experiment that requires participants to learn how to avoid adverse choices based on emotional feedback (losing or winning in a virtual situation) (28). In this study, IGT behavioral results showed that the iTBS and sham groups showed an upward trend in identifying favorable and unfavorable options throughout the experiment. This result suggests that as the experiment process advanced, more participants in both groups chose more favorable options, and their understanding of the IGT improved. In the iTBS group, after treatment, patients with schizophrenia began to realize that high-yield options are usually accompanied by high risks, so there is a significant difference in the scores before and after treatment in Block 1. However, patients have poor cognitive flexibility and it is difficult to adjust their attitudes toward risk decision-making; hence, while the increasing trend is not very obvious, as time goes by, the net score gradually increases. Therefore, the curve in the subsequent block shows a downward trend and then slowly rises. For the sham group, there was no significant change in the increase in scores. Although the performance of both groups was higher than that at baseline level after treatment, neither showed a clear advantage. The reason may be that the overall decision-making ability of patients with schizophrenia is poor and the learning curve for the IGT was steep (42, 43), and this particular group cannot catch the feedback stimulation processing in time in the IGT. In addition, patients with schizophrenia exhibit higher risk tolerance and tend to accept high punishments in exchange for immediate high returns. Their decision-making process follows the “immediate benefit first” strategy. This is also in line with Bechara's “short-sighted behavior,” which focuses on immediate interests and ignores long-term interests (44). The results of symptom-related analysis in this study showed that there was a correlation between the SANS score and the net score, and between the SANS score and the remaining money. This result indicates that the decision-making ability of patients with schizophrenia is affected by the severity of negative symptoms. The severity of negative symptoms is an important factor affecting the abnormal decision-making behavior of individuals with schizophrenia; the higher the level of negative symptoms, the more obvious the abnormal degree of decision-making behavior of individuals.

In the time-frequency domain, theta activity is generally related to the individual's processing of feedback stimuli, and it is sensitive to risk assessment in the process of decision-making processing (45). Based on the principle of time-frequency analysis, this study used the narrow band of 3–5 Hz (theta wave) as the analysis frequency band, and analyzed the energy change in theta waves in the 400–550 ms time window. The results of EROs showed that the iTBS group caused a larger range of theta activity than the sham group when the loss was selected under high-risk conditions at 400–550 ms after stimulation. This result indicates that the iTBS group has a significantly increased sensitivity to negative feedback after treatment. In other words, it proves that excitatory iTBS (5–Hz stimulus mixed with a 50–Hz high-frequency stimulus) can interfere with the abnormal decision-making behavior of patients with schizophrenia, which mainly manifests in the processing of negative feedback (32). In the IGT experimental paradigm adopted in this study, from a long-term perspective with the 100 Yuan option as the unfavorable option, the theta activity induced by its loss or gain is more intense. The sensitivity of schizophrenia to the loss of 100 Yuan increased after iTBS treatment. The reason for this may be that excitatory neurotransmitters mimic the explosive discharge of physiological action potentials in the central nervous system, further stimulating neurons in the prefrontal cortex, which may be particularly critical for regulating the risk-taking behavior (35). This is similar to the normal activity of the hippocampal neurons and can rapidly induce long-term enhancement. This induction phenomenon is similar to synaptic plasticity (46). Therefore, during continuous processing and learning of the feedback stimuli presented in the IGT task, the risk attitudes of participants gradually changed, and they became more sensitive to the damage caused by huge losses. The theta activity in the sham group did not show a significant effect, suggesting that its sensitivity to feedback stimuli and the learning effect of the regularity of tasks did not differ significantly.

In previous studies, the positive effects of iTBS on schizophrenia symptoms have been demonstrated. Chen and colleagues first used a patterned continuous theta burst stimulation suppression sequence in a 15-day continuous stimulation pattern to improve symptoms in patients with hallucinations (35). The results of subsequent studies conducted by Wang et al. proved the importance of prolonged iTBS stimulation in improving the symptoms of patients with schizophrenia (21). In the experiment, the design pattern of the personalized target and stereo frame navigation were used to monitor the position of the target in real time. This arrangement makes target positioning more precise and allows all stimuli to fall on the target setting in advance to minimize stimulus loss. In addition, the output intensity of all patients is based on their own RMT, and the treatment intensity is their optimal stimulation dose; hence, iTBS can maximize treatment results as much as possible.

This study has some limitations. First, there was no follow-up of participants after treatment; hence, the duration of the efficacy of TMS cannot be further determined. Second, our samples were relatively small, and the participants belonged to the same region in China. Then, there was a lack of healthy control groups in the study. Therefore, a large-scale study involving different regions may be needed in the future. Furthermore, the potential effect/bias of theta brainwave entrainment was also one of the limitations of this study, which requires further research to explore. Finally, our study is only an exploratory study with a small sample without detailed subcomponent types for schizophrenia.

## Conclusion

Our study is the first to demonstrate the importance of long-term iTBS in improving decision-making abilities in patients with schizophrenia. The results of our study provide evidence for improvements in iTBS-induced risk decision-making ability in schizophrenia. Our study indicates that iTBS as patterned TMS sequences had a specific benefit in schizophrenia with active treatment over sham treatment and a particular preponderance in improving risk decision-making.

## Data Availability Statement

The raw data supporting the conclusions of this article will be made available by the authors, without undue reservation.

## Ethics Statement

The studies involving human participants were reviewed and approved by Ethics Committee of Anhui Medical University. Written informed consent to participate in this study was provided by the participants' legal guardian/next of kin.

## Author Contributions

YW and LW performed the analysis and wrote the manuscript. GX, XF, LW, and ZC helped to collect the behavioral and ERSP data. FY and G-JJ helped in ERSP data analysis. CX and KW designed and supervised the study. All authors contributed to the article and approved the submitted version.

## Conflict of Interest

The authors declare that the research was conducted in the absence of any commercial or financial relationships that could be construed as a potential conflict of interest.
