# Supplementary material for: Intermittent Theta Burst Stimulation (iTBS) as an Optimal Treatment for Schizophrenia Risk Decision: an ERSP Study
Source: Front Psychiatry. 2021 May 10;12:594102. doi: 10.3389/fpsyt.2021.594102 (PMC8143028; doi:10.3389/fpsyt.2021.594102)
Supplement: Supplementary file 1 [file Data_Sheet_1.DOCX]

***Supplementary Materials***

**Results**

**Correlation between clinical characteristics and the revised Iowa Gambling Task**

We found a significant correlation between symptom severity and behavior (Supplementary Figure S3). Pearson’s correlation analysis showed that there was a highly significant negative correlation (r = 0.476, p = 0.063) between the SANS score and the net score in the independent testing agency group (Figure S3-A). In addition, we also observed that in the iTBS group, the SANS score was negatively correlated with the remaining money (r = 0.504, p = 0.047) (Figure S3-B). In the iTBS group, no significant relationship was found between spectral theta power and severity of symptoms.


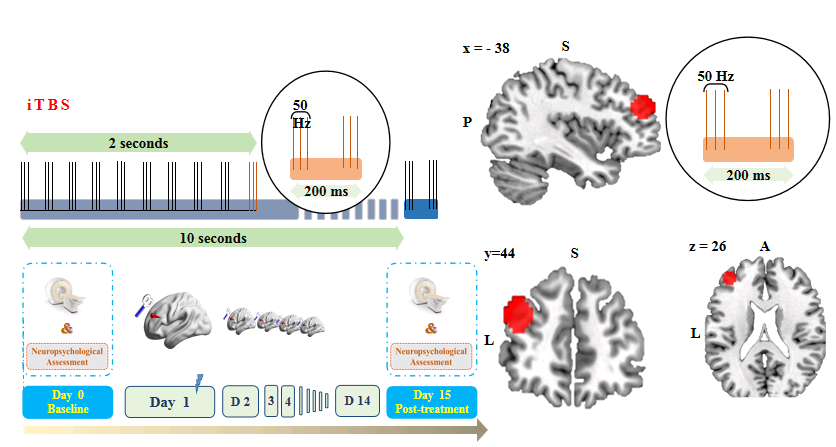


**Figure S1.** Summary of the experimental procedure and duration. The participants received neuropsychological assessments on the first and the last day. Continuous treatment was administered during the middle 14 days. The specific parameters of treatment of all participants and the location of the stimulation target were the same.


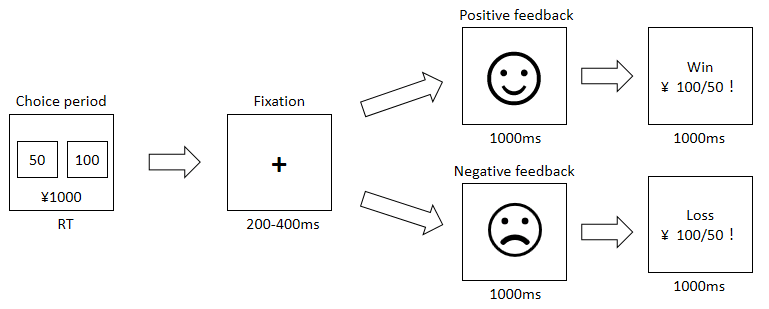


**Figure S2.** Schematic diagram in a single trial of the revised Iowa Gambling Task (IGT). RT, response time. In this example, the participants were presented with a choice of two alternatives, one of which they were asked to select by pressing a button using their left or right index finger. The presentation would remain until the button was pressed. After a fixation point appeared and lasted for 200–400 ms, the participants received feedback from a cartoon face for 1000 ms, indicating whether they lost or won in the trial. Subsequently, a numerical stimulus popped up on the computer screen to indicate the selected consequence, which lasted for 1000 ms.


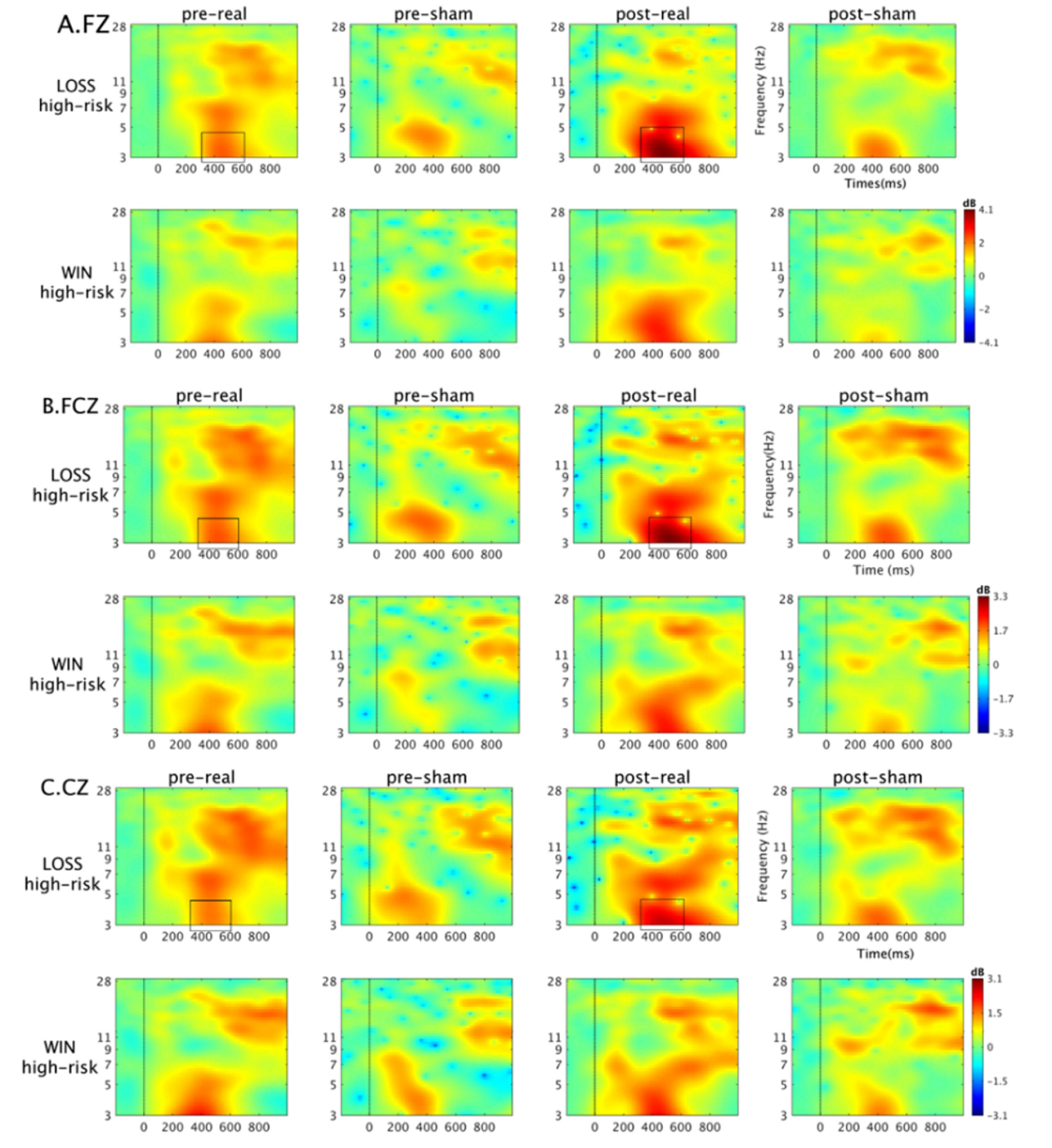


**Figure S3.** Event-related spectral perturbation (ERSP) results in the intermittent theta burst stimulation (iTBS) and sham groups based on channel electroencephalography data. The results obtained from the FZ, FCZ and CZ electrodes are shown in Figure A-C. The black boxes define the time-frequency region of interest where the power increases significantly.


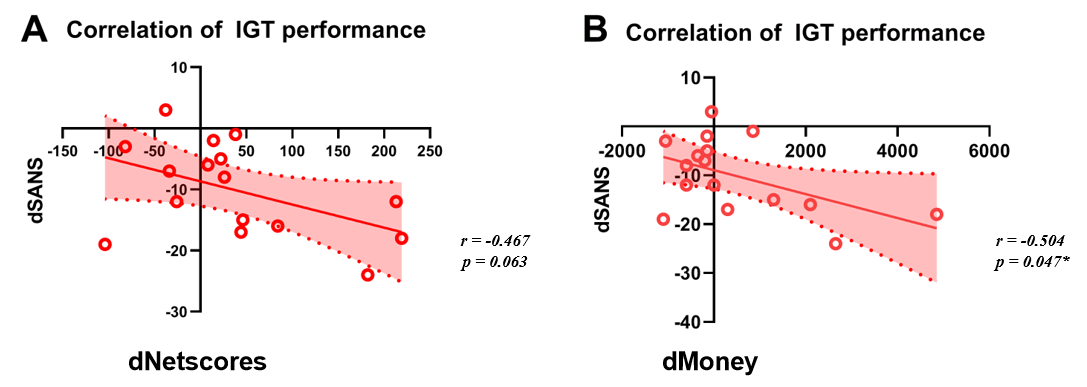


**Figure S4.** Correlation between revised Iowa Gambling Task (IGT) performance and Scale for the Assessment of Negative Symptoms (SANS) scores. A scatter plot with Pearson’s correlation analysis shows the relationship between the total net scores and SANS (A), and the correlation between the final amount of money and SANS (B). *** *p* < 0.001; ** *p* < 0.01; * *p* < 0.05; ns: *p* > 0.05.

**Table S1.** **Planned 2 × 2 repeated measure ANOVA on ERP over time (pre-TMS, post-TMS)**

|  | iTBS treatment  (n=16) | | Sham treatment  (N=25) | | Factor Time | | Group by time  interaction | | |
| --- | --- | --- | --- | --- | --- | --- | --- | --- | --- |
|  | Pre | Post | Pre | Post | F | *p*^b^ | F | *p*^b^ | *Effect Sizes*^c^ |
| **IGT** |  |  |  |  |  |  |  |  |  |
| Netchoice | 28.69(82.44) | 66.94(118.58) | 12.13(60.71) | 63.13(135.83) | 5.146 | *0.031** | 0.105 | 0.748 | 0.003 |
| Money | 121.88(1683.94) | 609.38(1700.61) | -428.13(1793.32) | 746.88(2142.31) | 9.027 | *0.005*** | 1.544 | 0.224 | 0.049 |
| **ERP** |  |  |  |  |  |  |  |  |  |
| FPZ100 | 2.28(1.95) | 4.46(4.34) | 1.43(1.67) | 2.23(3.86) | 6.243 | *0.018** | 1.336 | 0.257 | 0.043 |
| FPZ101 | 1.53(2.90) | 3.02(4.583) | 0.67(1.89) | 1.03(3.91) | 2.015 | 0.166 | 0.747 | 0.394 | 0.024 |
| FPZ50 | 2.17(1.87) | 2.39(3.56) | 1.31(1.88) | 1.73(3.00) | 0.235 | 0.631 | 0.022 | 0.882 | 0.001 |
| FPZ51 | 1.10(2.27) | 1.65(4.08) | 0.45(1.83) | 1.44(3.07) | 0.972 | 0.332 | 0.080 | 0.780 | 0.003 |
| FZ100 | 2.15(1.81) | 3.85(3.48) | 1.28(1.53) | 1.68(2.79) | 5.487 | *0.026** | 2.118 | 0.156 | 0.066 |
| FZ101 | 1.46(2.64) | 2.69(3.88) | 0.46(1.43) | 0.70(2.56) | 2.098 | 0.158 | 0.973 | 0.332 | 0.031 |
| FZ50 | 2.14(1.57) | 2.37(2.82) | 1.19(1.68) | 1.36(2.20) | 0.166 | 0.686 | 0.004 | 0.949 | 0.000 |
| FZ51 | 0.97(1.73) | 1.47(3.25) | 0.59(.95) | 0.91(1.87) | 0.530 | 0.472 | 0.028 | 0.868 | 0.001 |
| FCZ100 | 1.90(1.71) | 3.04(2.35) | 1.10(1.36) | 1.58(2.30) | 6.455 | *0.016** | 1.074 | 0.308 | 0.035 |
| FCZ101 | 1.40(2.47) | 2.12(2.48) | 0.57(1.41) | 0.82(1.93) | 1.856 | 0.183 | 0.429 | 0.517 | 0.014 |
| FCZ50 | 1.70(1.22) | 2.09(1.74) | 1.02(1.50) | 1.15(1.80) | 0.592 | 0.448 | 0.143 | 0.708 | 0.005 |
| FCZ51 | 0.96(1.66) | 1.26(2.31) | 0.53(0.93) | 0.78(1.55) | 0.381 | 0.542 | 0.003 | 0.958 | 0.000 |
| CZ100 | 1.20(0.92) | 2.31(1.89) | 0.72(1.00) | 1.30(1.89) | 9.410 | *0.005*** | 0.874 | 0.357 | 0.028 |
| CZ101 | 1.56(2.23) | 1.84(1.89) | 0.67(1.45) | 0.91(1.67) | 0.734 | 0.398 | 0.004 | 0.949 | 0.000 |
| CZ50 | 1.37(1.19) | 1.64(1.51) | 0.67(1.27) | 0.94(1.39) | 0.958 | 0.335 | 0.000 | 0.995 | 0.000 |
| CZ51 | 1.08(1.54) | 1.28(2.13) | 0.56(0.67) | 0.94(1.52) | 0.470 | 0.498 | 0.044 | 0.835 | 0.001 |

*Note*:

a. Two-sample t test between pre- real and pre- sham TMS treatment;

b. Group by time interaction effect by repeated measures ANOVA.

c. Effect sizes for the interaction between group and time of measurement were calculated by subtracting the mean score post treatment from the mean score before treatment for each group, subsequently determining the difference between the 2 groups (iTBS, sham) and then dividing the results by the pooled SDs

^*^ *p*<0.05

^**^ *p*<0.01

^***^ *p*<0.001
